# Improved Risk Stratification Prior to Major Pulmonary Resection by Combining Peak Oxygen Uptake and Ventilatory Efficiency in a 9-Field Matrix

**DOI:** 10.1016/j.chpulm.2025.100192

**Published:** 2025-07-24

**Authors:** Karolina Kristenson, Johan Hylander, Miklós Boros, Éva Tamás, Gabriel Högström, Kristofer Hedman

**Affiliations:** a Department of Cardiothoracic and Vascular Surgery in Linköping, and Department of Health, Medicine and Caring Sciences, Linköping University, Linköping, Sweden; b Department of Anesthesiology and Intensive Care in Linköping, and Department of Biomedical and Clinical Sciences, Linköping University, Linköping, Sweden; c Department of Pulmonology in Linköping, and Department of Health, Medicine and Caring Sciences, Linköping University, Linköping, Sweden; d Department of Clinical Physiology, and Department of Health, Medicine and Caring Sciences, Linköping University, Linköping, Sweden

**Keywords:** CPET, lobectomy, pulmectomy, surgery, $\dot{V}E$/Vco_2_ slope, $\dot{V}o_{2}$

## Abstract

**Background:**

Cardiopulmonary exercise testing (CPET) has a pivotal role in preoperative evaluation of patients before lung cancer surgery. As surgical and perioperative practice and functional diagnostics continuously evolve, it may be time to reevaluate and refine the use of CPET in this setting.

**Research Question:**

Can risk assessment with CPET before lung cancer surgery be improved by combining 2 established CPET variables (percent predicted peak oxygen uptake [Vo_2_peak], and ventilatory efficiency, measured by minute ventilation [$\dot{V}E$]/carbon dioxide elimination [Vco_2_] slope) while using recently suggested optimal threshold values for these variables?

**Study Design and Methods:**

Single-center, retrospective analysis of 208 patients with lung cancer who underwent preoperative CPET in 2008 to 2020. The main outcome was any major pulmonary complication (MPC) or death within 30 days of surgery. We combined previously suggested threshold values of percent predicted Vo_2_peak and $\dot{V}E$/Vco_2_ slope, defined with a focus on high sensitivity and specificity. For each measure, patients were categorized into 3 groups based on these thresholds, yielding a proposed 9-field matrix for risk assessment. The frequency of complications between groups was compared using the χ^2^ test.

**Results:**

Overall, 29 patients (14%) suffered an MPC and 3 died. The frequency of complications differed between groups based on the 9-field matrix in patients who underwent lobectomy or pulmectomy (*P* < .001). No patient with both favorable percent predicted Vo_2_peak and $\dot{V}E$/Vco_2_ slope values experienced MPC or death, whereas worsening values in both percent predicted Vo_2_peak and $\dot{V}E$/Vco_2_ slope were associated with an increasing frequency of adverse outcomes.

**Interpretation:**

The proposed 9-field matrix for risk assessment was able to demonstrate a synergistic effect between $\dot{V}E$/Vco_2_ slope and percent predicted Vo_2_peak for identifying patients who suffered major pulmonary complications or death within 30 days of cancer lobectomy or pulmectomy. These results further improve and help nuance risk assessment in these patients.

**Take-Home Points**

**Study Question:** Can the preoperative risk assessment with cardiopulmonary exercise testing before major lung cancer surgery be improved by using a combination of 2 well-established cardiopulmonary exercise testing variables (percent predicted peak oxygen uptake and ventilatory efficiency [as measured by minute ventilation/carbon dioxide elimination slope]) and applying recently suggested optimal threshold values for each variable?

**Results:** The proposed 9-field matrix for risk assessment was able to identify a synergistic effect between minute ventilation/carbon dioxide elimination slope and percent predicted peak oxygen uptake to detect major pulmonary complications or death within 30 days of cancer lobectomy or pulmectomy.

**Interpretation:** The proposed 9-field matrix could support clinicians in further improving and nuancing risk assessment before lung cancer surgery.

Cardiopulmonary exercise testing (CPET) is considered the criterion standard for assessing functional capacity as part of risk stratification in candidates for major pulmonary resection.^1^ Current international guidelines identify patients with a peak oxygen consumption (Vo_2_peak) < 10 mL/kg/min as high risk, patients with a Vo_2_peak of 10 to 20 mL/kg/min as moderate risk, and those with a Vo_2_peak > 20 mL/kg/min as low risk of perioperative complications or death.^2^^,^^3^ However, surgical and perioperative practice continuously evolve, and current guidelines (published in 2009^2^ and 2013,^3^ respectively) are based mainly on data from studies dated 15 to 25 years ago. It is possible that the proposed thresholds are not optimal today.^4^ Moreover, in addition to Vo_2_peak, CPET also provides measures of ventilatory efficiency, such as the slope of the increase in minute ventilation ($\dot{V}E$) to carbon dioxide elimination (Vco_2_). In the last decade, several studies on preoperative evaluation before lung cancer surgery have found an association between $\dot{V}E$/Vco_2_ slope and mortality,^5^, ^6^, ^7^, ^8^, ^9^ and with perioperative pulmonary complications.^8^, ^9^, ^10^, ^11^

An algorithm for preoperative stratification of patients’ risk of perioperative complications that incorporates Ve/Vco_2_ slope has been proposed by Salati and Brunelli.^1^ They suggest a threshold value of 35 to further risk stratify patients in the moderate risk group (Vo_2_peak of 10-20 mL/kg/min) as intermediate-low or intermediate-high, respectively.^1^ Recently, studies have showed that risk assessment could be further improved (1) by assessing Vo_2_peak in relation to normative values also accounting for age and sex (percent predicted), rather than only considering body weight^12^; and (2) by assessing $\dot{V}E$/Vco_2_ slope based on 3 rather than 2 risk categories (ie, 2 thresholds rather than a single value).^13^ Together, this indicates that a combined assessment of Vo_2_peak (percent predicted) and $\dot{V}E$/Vco_2_ slope, each categorized into 3 risk groups, could provide an improved assessment of perioperative risk in lung cancer surgery.

Therefore, in this study we aimed to refine the preoperative risk assessment with CPET before major lung cancer resection by (1) using a combination of percent predicted Vo_2_peak and ventilatory efficiency ($\dot{V}E$/Vco_2_ slope) and (2) applying recently defined optimal threshold values for both of these variables.

Our hypothesis was that frequencies of complications within the 9-field matrix were significantly different between groups (evaluated with χ^2^ testing), which would allow detection of a synergistic effect between $\dot{V}E$/Vco_2_ slope and percent predicted Vo_2_peak to predict major pulmonary complications (MPCs) or death within 30 days of major lung cancer surgery.

## Study Design and Methods

This was a longitudinal cohort study using retrospectively collected, manually analyzed CPET data and with prospective collection of outcome data from national registries. It included all patients with lung cancer who underwent pneumectomy, lobectomy, or sublobular resection and preoperative CPET during the years 2008 to 2020 at a single center (Linköping University Hospital, Sweden). Because sublobular resections are not included in the algorithm for preoperative physiological evaluation in current US guidelines,^3^ we performed separate analyses for patients who underwent pulmectomy/lobectomy and sublobular resections. Ethical permission was granted by the Swedish Ethical Review Authority (Dnr 2020-03375, 2020-05284, 2021-00543).

### Outcome Definition and Registration

The main outcome was any MPC or death within 30 days from surgery, where MPC was defined as any pneumonia, pulmonary embolus, empyema, delayed extubation (not able to extubate in the operation room directly after surgery), reintubation, reoperation, ARDS, respiratory insufficiency, or pulmonary edema. Definitions of complications and comorbidities harmonize with international guidelines.^14^

CPET data were cross linked with 3 separate databases using the Swedish social security number. First, the Swedish National Quality Register for General Thoracic Surgery^15^ was used to retrieve data on in-hospital complications, comorbidities, operation code, and surgical technique (open approach or minimally invasive thoracic surgery). Data were then cross linked with the Swedish National Patient Register,^16^ containing all inpatient and outpatient hospital diagnoses of each Swedish citizen. Finally, the Swedish Cause of Death Register was used to determine the survival status and date of death.^16^ More detailed information about these Swedish registries is presented in the supplementary data 1.

### Cardiopulmonary Exercise Testing

Maximal CPET was performed on a cycle ergometer, including 5 minutes of warm-up at 10 to 50 W, followed by a continuous incremental ramp protocol of 10 to 20 W/min (eBike Basic; GE Medical Systems). The warm-up and incremental workloads were individualized for each patient, aiming to reach exhaustion after 8 to 12 minutes of exercise. Patient monitoring included systolic BP, electrocardiogram (Marquette CASE 8000; GE Medical Systems), rating of perceived exertion (Borg RPE scale), chest pain, and dyspnea (Borg CR-10 scale).^17^

Gas exchange and ventilatory variables were analyzed on a breath-by-breath basis (Jaeger Oxycon Pro or Vyntus CPX; Viasys Healthcare) with a system calibrated before each test. Oxygen uptake, Vco_2_, and $\dot{V}E$ were presented numerically as 10-second means, excluding breaths with the highest and lowest values. Vo_2_peak was defined as the average of the 2 highest consecutive 10-second mean oxygen uptake values at or close to the end of the exercise, and was presented as weight-indexed Vo_2_peak (divided by body mass, mL/kg/min) and as percent predicted in relation to predicted Vo_2_peak values calculated from reference equations presented in the SHIP study (percent predicted Vo_2_peak).^18^ The $\dot{V}E$/Vco_2_ slope was manually measured up until the respiratory compensation point using commercially available software (Sentry Suite 3.10; CareFusion GmbH).

### Nine-Field Matrix and Risk Groups

To construct a 9-field matrix for risk stratification, threshold values for percent predicted Vo_2_peak and $\dot{V}E$/Vco_2_ slope were chosen based on previous research, where receiver operating characteristic analysis was used to define a lower 90% sensitivity (percent predicted Vo_2_peak > 88%, $\dot{V}E$/Vco_2_ slope ≤ 30) and an upper 90% specificity threshold (percent predicted Vo_2_peak < 62%, $\dot{V}E$/Vco_2_ slope > 40) for each measure, in relation to the main outcome.^12^^,^^13^ Then, based on these thresholds, patients were categorized into 1 of 3 groups for each of the 2 measures, yielding a 9-field matrix (3 × 3 possible combinations of categories for percent predicted Vo_2_peak and $\dot{V}E$/Vco_2_ slope).

In addition, patients were divided into 4 groups based on what we defined would be a clinically relevant stratification in frequency of complications: very high (> 50% MPCs or death), high (25%-50%), intermediate (15%-24%), and low risk (< 15%).

### Pulmonary Function Testing

Preoperative pulmonary function testing included static and dynamic lung volumes (FEV_1_, FVC, total lung capacity, and residual volume) and carbon monoxide lung diffusion capacity corrected for hemoglobin (Dlcoc). Pulmonary function data were expressed as crude values and percent predicted values.^19^^,^^20^

### Statistics

Cross linking of databases and database management were performed using R Studio v1.1.456 (R Studio Inc). Statistical analyses were performed with SPSS 29.0.1.1 (244) (SPSS Inc). Frequencies of complications within the 9-field matrix and presence of comorbidities across different operation types or risk groups were compared with a χ^2^ test. The χ^2^ test was further used to estimate whether there was a statistically significant difference between the expected frequencies and the observed frequencies in ≥ 1 categories of a contingency table. Mean values of continuous data from basic patient characteristics (age, height, and weight) and CPET and spirometry data were compared with the Student *t* test. The significance level was set to *P* < .05 and was tested 2-sidedly.

To estimate different models’ ability to predict the main outcome, the area under the curve (AUC) from a receiver operating curve analysis was determined and presented with a 95% CI for (1) the 4 risk groups visualized in the 9-field matrix, (2) the 3 risk groups based on percent predicted Vo_2_peak only, (3) the 3 risk groups based on $\dot{V}E$/Vco_2_ slope only, and (4) the 4 risk groups previously suggested by Salati and Brunelli.^1^

We performed 2 sensitivity analyses. First, to study if including only truly maximal exercise tests would impact on our results, we performed a sensitivity analysis where we excluded patients with a respiratory exchange ratio < 1.05 in combination with either < 85% predicted maximal heart rate (and without beta-blocker medication) or a breathing reserve > 30%. In a second sensitivity analysis, we evaluated if our results were valid when excluding patients with normal preoperative pulmonary function, by excluding patients with both FEV_1_ and Dlcoc > 80% predicted values.^2^

## Results

A total of 208 patients (97 men [47%]; mean age, 71 ± 7 years) with a pathologic-anatomic diagnosis of lung cancer who had undergone pulmectomy (n = 20), lobectomy (n = 138, including 10 bilobectomies), or sublobular resection (n = 50), and for whom there were available data on preoperative Vo_2_peak and $\dot{V}E$/Vco_2_ slope, were included.

For all patients, mean weight-indexed Vo_2_peak was 18 ± 4 mL/kg/min (range, 10-31), percent predicted Vo_2_peak was 82% ± 16% (range, 45-117), and $\dot{V}E$/Vco_2_ slope was 34 ± 6 (range, 19-58). Data per type of lung surgery are presented in Table 1.

**Table 1:** Study Cohort Characteristics

	Pulmectomy			Lobectomy			Sublobular Resection		
	No.	Mean	[SD]	No.	Mean	[SD]	No.	Mean	[SD]
**Basic Characteristics**									
Male, No. (%)	17 (85)			58 (42)			22 (44)		
Age, y	20	68.0	[5.1]	138	70.9	[7.8]	50	73.6	[5.8]
Height, cm	20	176.7	[7.3]	138	168.2	[9.0]	50	169.6	[8.7]
Weight, kg	20	75.7	[13.4]	138	75.5	[16.3]	50	76.1	[18.9]
BMI, kg/m^2^	20	24.1	[3.5]	138	26.6	[4.9]	50	26.3	[5.8]
CPET									
Vo_2_peak, mL/kg/min	20	20.4	[4.3]	138	17.5	[3.8]	50	17.7	[4.1]
Vo_2_peak % predicted	20	77.9	[15.7]	138	81.4	[15.2]	50	84.9	[16.7]
$\dot{V}E$/Vco_2_ slope	20	32.0	[5.3]	138	34.0	[6.4]	50	34.4	[6.4]
Spirometry									
FEV_1_, L/min	20	2.3	[0.5]	138	2.1	[0.7]	50	2.0	[0.7]
FEV_1_ % predicted	20	68.0	[13.2]	138	77.3	[19.6]	50	76.6	[20.2]
VC, L	20	3.9	[0.9]	138	3.3	[0.9]	50	3.4	[0.9]
FVC % predicted	20	80.7	[18.4]	138	70.7	[21.3]	50	73.9	[26.5]
FEV_1_/VC	20	0.6	[0.1]	138	0.6	[0.1]	50	0.6	[0.1]
Dlcoc, mmol/min/kPa	18	5.9	[1.1]	118	5.5	[1.7]	43	5.0	[1.7]
Dlcoc % predicted	18	71.6	[12.3]	118	79.0	[19.9]	43	74.9	[24.3]
TLC, L	18	7.1	[1.6]	120	6.1	[1.2]	41	6.3	[1.3]
TLC % predicted	18	94.9	[15.6]	120	98.4	[15.0]	41	100.8	[13.5]
RV, L	18	3.1	[0.8]	118	2.6	[0.7]	41	2.9	[0.8]
RV % predicted	18	117.6	[27.3]	118	114.1	[34.9]	41	122.1	[33.0]
**Comorbidity**	**No.**	**%**		**No.**	**%**		**No.**	**%**	
Coronary artery disease	3	15		18	13		8	16	
Previous cardiac surgery	1	5		12	9		3	6	
Previous cerebrovascular insult	1	5		12	9		3	6	
Current treatment for heart failure	1	5		9	7		3	6	
Current treatment for hypertension	5	25		54	39		19	38	
Current treatment for arrhythmia	2	10		12	9		7	14	
Diabetes mellitus	0	0		28	20		12	24	
Chronic kidney disease	1	5		8	6		4	8	
COPD	6	30		55	40		21	42	
MITS	0	0		15	11		7	14	

CPET = cardiopulmonary exercise testing; Dlcoc = diffusing capacity of the lungs for carbon monoxide, MITS = minimally invasive thoracic surgery; RV = residual volume; TLC = total lung capacity; VC = vital capacity; Vco_2_ = carbon dioxide elimination; $\dot{V}E$ = minute ventilation; Vo_2_peak = peak oxygen uptake.

Within 30 days after surgery, 29 patients (14%) experienced MPCs, of which 3 had died. The frequency of MPCs or death was 6% for patients who underwent sublobular resection, 17% for lobectomy, and 15% for pulmectomy. There was no difference in the frequency of MPCs or death based on surgical technique (minimally invasive thoracic surgery: 14% vs open approach: 14%, *P* = .97).

### Nine-Field Matrix

**Figure 1:**
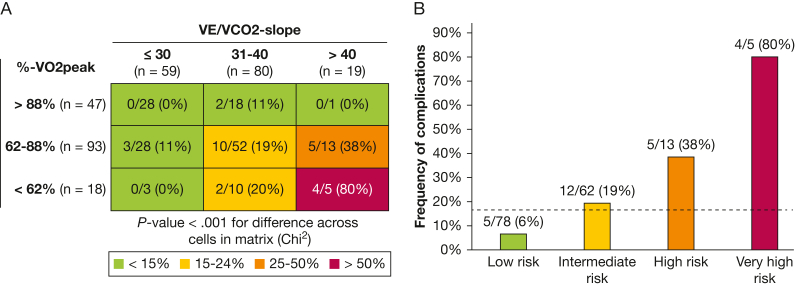
Proportion of major pulmonary complications or death within 30 days of lung cancer surgery (lobectomy or pulmectomy) per category of precent predicted peak oxygen uptake (%-VO2peak) and ventilatory efficiency (VE/VCO2-slope) in 156 patients.

The frequency of complications differed between the 9 groups of the 9-field matrix for patients who underwent either pulmectomy or lobectomy (n = 158, *P* < .001) (Fig 1A). Importantly, in these patients, those with low (< 15% MPCs or death), intermediate (15%-24%), high (25%-50%), and very high risk (> 50%) could be identified when combining the 2 measures and proposed thresholds (Fig 1B). In contrast, for patients who underwent sublobular resection (n = 50), no relation with outcome was found when applying the proposed 9-field matrix (*P* = .56) (e-Fig 1C). Further analyses therefore include only the 158 patients who underwent either lobectomy or pulmectomy. Separate analyses for lobectomy and pulmectomy are listed in e-Figure 1A and 1B. Patient characteristics based on 4 risk groups visualized in the 9-field matrix are presented in e-Table 1.

AUC values (with 95% CI) for different methods for risk categorization are presented in Table 2.

**Table 2:** AUC Values From Receiver Operating Curve Analyses Using Different Methods to Risk Stratify Candidates to Major Lung Cancer Resection

Method of risk stratification	AUC	SE	*P* Value	95% CI
Four groups visualized in 9-cell matrix^a^	0.73	0.06	< .001	0.62-0.84
Three groups from % predicted Vo_2_peak^b^	0.67	0.05	.002	0.56-0.77
Three groups from $\dot{V}E$/Vco_2_ slope^c^	0.70	0.06	< .001	0.59-0.81
Four groups previously proposed algorithm^d^	0.67	0.60	.005	0.55-0.79

AUC = area under the curve; Vco_2_ = carbon dioxide elimination; $\dot{V}E$ = minute ventilation; Vo_2_peak = peak oxygen uptake.

a Low risk = $\dot{V}E$/Vco_2_ slope ≤ 30 or % predicted Vo_2_peak > 88%, intermediate risk = $\dot{V}E$/Vco_2_ slope 31-40 and % predicted Vo_2_peak ≤ 88%, high risk = $\dot{V}E$/Vco_2_ slope > 40 and % predicted Vo_2_peak 62%-88%, and very high risk = $\dot{V}E$/Vco_2_ slope > 40 and % predicted Vo_2_peak < 62%.

b Low risk = % predicted Vo_2_peak > 88%, intermediate risk = % predicted Vo_2_peak 62%-88%, and high risk = % predicted Vo_2_peak < 62%.

c Low risk = $\dot{V}E$/Vco_2_ slope ≤ 30, intermediate risk = $\dot{V}E$/Vco_2_ slope 31-40, and high risk = $\dot{V}E$/Vco_2_ slope > 40.

d Low risk = Vo_2_peak > 20 mL/kg/min, intermediate/high risk = Vo_2_peak 10-20 mL/kg/min and $\dot{V}E$/Vco_2_ slope > 35, intermediate/low risk = Vo_2_peak 10-20 mL/kg/min and $\dot{V}E$/Vco_2_ slope ≤ 35, and high risk = Vo_2_peak < 10 mL/kg/min.

We performed 2 sensitivity analyses. First, when excluding 26 patients with possibly nonmaximal CPET, similar results were found in the 9-field matrix (e-Fig 2A). Second, when excluding 38 patients with normal lung function (both percent predicted FEV_1_ and percent predicted Dlcoc > 80%), very similar results were found in the 9-field matrix (e-Fig 2B).

## Discussion

This study of 208 patients who underwent CPET before lung cancer surgery suggests that preoperative risk assessment can be further improved in patients undergoing lobectomy or pulmectomy, by combining percent predicted Vo_2_peak and $\dot{V}E$/Vco_2_ slope into a 9-field matrix (Fig 1).

Importantly, our results underscore the value of using a combination of both Vo_2_peak and $\dot{V}E$/Vco_2_ slope in risk assessment, and not to oversimplify risk estimation by using single threshold values for either parameter. As apparent from the proposed 9-field matrix, patients had a gradually and synergistic increased risk of complications or death as CPET parameters worsened. For example, none of the 28 patients with low-risk values for both percent predicted Vo_2_peak and $\dot{V}E$/Vco_2_ slope suffered an MPC or death, thus identifying a group of patients at very low risk. At the other end of the spectrum, patients with highly abnormal values for both percent predicted Vo_2_peak and $\dot{V}E$/Vco_2_ slope had a very high risk of complications, reaching a frequency of MPC or death of 80%.

In contrast, the 9-field matrix was not useful for risk stratification in patients with sublobular resection. In this study, sublobular resection was overall associated with a low risk of complications, in magnitude similar to the risk seen in patients undergoing lobectomy or pulmectomy who had a low risk according to the 9-field matrix. Current guidelines do not include sublobular resections in the CPET-based risk assessment,^3^ which the results from the current study support. However, it is important to acknowledge that also in the sublobular patient group, no patient within this cohort had a Vo_2_peak < 10 mL/kg/min. This study used percent predicted Vo_2_peak values calculated from reference equations presented in the SHIP study.^18^ If another reference equation for percent predicted Vo_2_peak is used, slightly different threshold values may apply.^12^

We also showed that it is uncommon that a patient present with a combination of CPET measures (percent predicted Vo_2_peak and $\dot{V}E$/Vco_2_ slope) where 1 is categorized as high risk and the other is categorized as low risk. In the current population, only 4 patients (2%) had such a combination, and 2 of these patients were excluded when applying strict criteria for true maximal CPET. This suggest that such a combination of CPET measures should warrant a particular quality assessment and, possibly, repeated CPET.

### $\dot{V}E$**/**Vco_2_ Slope in Risk Assessment

$\dot{V}E$/Vco_2_ slope has evolved as a strong marker of risk within many areas of medicine. It has been associated with negative events in pulmonary arterial hypertension and heart failure,^21^, ^22^, ^23^ and in the last decade studies on preoperative evaluation for lung surgery have found a stronger association between $\dot{V}E$/Vco_2_ slope and mortality and morbidity, compared with Vo_2_peak.^5^, ^6^, ^7^, ^8^, ^9^

Previous incorporation of $\dot{V}E$/Vco_2_ slope to improve risk stratification in patients with intermediate values of Vo_2_peak has been suggested not only for patients with lung cancer^1^^,^^24^ but also in the preoperative evaluation for esophagus cancer,^25^ for major abdominal cancer,^26^ and for prognostication in heart failure.^27^ To our knowledge, this is the first study that combines thresholds derived with the purpose to establish true low-risk and high-risk individuals, using both of these 2 CPET measures. When combining these 2 measures, a higher AUC value was found, compared with either separate models for Vo_2_peak or $\dot{V}E$/Vco_2_ slope, but also compared with using 3 risk categories of Vo_2_peak (< 10, 10-20, or > 20 mL/kg/min, respectively) in combination with a single threshold for $\dot{V}E$/Vco_2_ slope set at 35.^1^^,^^28^ Although this in part may be attributed to the fact that percent predicted Vo_2_peak may be superior to using weight-indexed Vo_2_peak,^12^ our results also suggest that important prognostic information may be lost when dichotomizing $\dot{V}E$/Vco_2_ slope at 35. Overall, our results underscore the importance of ventilatory efficiency and how CPET can be leveraged in preoperative evaluation.

### Strengths and Limitations

The strengths of this study include the relatively large and homogenous sample of patients, and the methodology where we combine threshold values of percent predicted Vo_2_peak and $\dot{V}E$/Vco_2_ slope, established to determine a priori-defined levels of sensitivity and specificity instead of choosing arbitrary values to define thresholds. This offers clinicians a nuanced risk stratification, derived by a robust methodologic approach. The 9-field matrix was found to be insensitive to nonmaximal exercise tests and to preoperative spirometry data. Finally, by using the Swedish social security number in combination with 2 national Swedish registries of known high quality to define the occurrence of complications, there was no loss to follow-up, and because only MPCs or death were included as outcomes, the risk of underreporting in the registries is minimal.

This study is limited by the single-center design, and our results need validation in a second cohort, preferably including a wider selection of ethnic groups. Also, this study was conducted at a center with a very low mortality for patients undergoing lung cancer surgery.^15^ Therefore, the generalizability to other settings is unknown. We were also unable to include patients with very low Vo_2_peak (ie, < 10 mL/kg/min) because these patients were excluded from surgery based on the well-established, very high risk of complications associated with such a low Vo_2_peak,^29^, ^30^, ^31^ in accordance with current international guidelines.^2^^,^^3^ Therefore, the thresholds proposed in the current study are only fully valid when excluding patients with Vo_2_peak < 10 mL/kg/min. The 9-field matrix approach led to quite small subgroups for some of the combinations of CPET variables, especially for the very high risk group. Therefore, the point estimates within risk groups with a limited sample should be evaluated with caution. Also, even though we have registered known comorbidities and spirometry data, the etiology for ventilatory efficiency in specific patients cannot be determined in this study. It could be caused by the lung cancer itself, underlying COPD with corresponding ventilation-perfusion mismatch, undiagnosed heart failure, or pulmonary arterial hypertension. In addition, this study only included complications or deaths that occurred within 30 days after surgery. Future studies should address if the risk groups identified in this study also are valid for intermediate-term and long-term mortality. Finally, 1 limitation of CPET is its availability, especially in low-income countries and at smaller surgical centers. Future studies should therefore—parallel to further improving the CPET method—strive to develop reliable and valid low technologic tests, especially to evaluate ventilatory efficiency.

## Interpretation

The proposed 9-field matrix for risk assessment was able to demonstrate a synergistic effect between $\dot{V}E$/Vco_2_ slope and percent predicted Vo_2_peak for identifying patients who suffered MPCs or death within 30 days of cancer lobectomy or pulmectomy. These results further improve and help nuance risk assessment in these patients.

## Funding/Support

This study was funded by unrestricted grants from ALF, 10.13039/100016670Region Östergötland, Sweden.

## Financial/Nonfinancial Disclosures

None declared.

## Supplementary Data

e-Online Data

e-Online Data

e-Online Data
