# Supplementary material for: Improved Risk Stratification Prior to Major Pulmonary Resection by Combining Peak Oxygen Uptake and Ventilatory Efficiency in a 9-Field Matrix
Source: CHEST Pulm. 2025 Jul 24;3(4):100192. doi: 10.1016/j.chpulm.2025.100192 (PMC13418083; doi:10.1016/j.chpulm.2025.100192)
Supplement: e-Online Data [file mmc1.docx]

**Supplementary data**

*Swedish patient registries*

The Swedish Quality Registry for General Thoracic Surgery has been active since 2008 and all eight thoracic surgery centers in Sweden take active part in the registration of patients since 2013. The data is registered online by the surgeon who manually enters each minor and major complication during the stay at hospital as well as other relevant factors such as surgical technique and patient comorbidities. Quality control for lung cancer patients, has revealed that 93% to 97% of patients were registered in the system nationwide between the years 2013-2019.(1)

The Swedish social security number (unique for every Swedish citizen) in combination with several nation-wide medical databases, which are mandatory to report all medical in-hospital diagnoses into, underlies the possibility to retrospectively study associations between almost every baseline condition and outcome in Sweden. Data is automatically and monthly or annually fetched from all medical journals and transferred to the National Board of Health, who after quality control and governance adds this to the register. It is thus highly unlikely that diagnoses of complications entered in the medical record are not available in the registries.(2) Most complications are thus “double coded” in both the National, governmental registries as well as in the Swedish Quality Registry for General Thoracic Surgery, which increases the validity of the completeness in the data collection in this study.

References

1. The Swedish National Quality Register for General Thoracic Surgery [Internet]. 2023 [cited 20230414]. Available from: <http://www.ucr.uu.se/thor>.

2. Ludvigsson JF, Andersson E, Ekbom A, Feychting M, Kim JL, Reuterwall C, et al. External review and validation of the Swedish national inpatient register. BMC Public Health. 2011;11:450.
