# Supplementary material for: Improved Risk Stratification Prior to Major Pulmonary Resection by Combining Peak Oxygen Uptake and Ventilatory Efficiency in a 9-Field Matrix
Source: CHEST Pulm. 2025 Jul 24;3(4):100192. doi: 10.1016/j.chpulm.2025.100192 (PMC13418083; doi:10.1016/j.chpulm.2025.100192)
Supplement: e-Online Data [file mmc2.docx]

**E-Tables**

**e-Table 1.** Patient characteristics based on four risk groups.

|  | **Low risk** | | |  | **Intermediate risk** | | |  | **High risk** | | |  | **Very high risk** | | |
| --- | --- | --- | --- | --- | --- | --- | --- | --- | --- | --- | --- | --- | --- | --- | --- |
|  | **N** | **Mean** | **SD** |  | **N** | **Mean** | **SD** |  | **N** | **Mean** | **SD** |  | **N** | **Mean** | **SD** |
| **Basic Characteristics** |  |  |  |  |  |  |  |  |  |  |  |  |  |  |  |
| Male, n (%) | 40 (36) |  |  |  | 40 (55) |  |  |  | 11 (58) |  |  |  | 6 (100) |  |  |
| Age, years | 110 | 71.3 | 7.6 |  | 73 | 70.5 | 7.3 |  | 19 | 74.8 | 5.2 |  | 6 | 69.8 | 3.6 |
| Height, centimeters | 110 | 167.9 | 8.7 |  | 73 | 171.5 | 9.5 |  | 19 | 169.9 | 9.2 |  | 6 | 170.3 | 3.4 |
| Weight, kg | 110 | 74.9 | 16.3 |  | 73 | 77.7 | 17.5 |  | 19 | 75.6 | 16.1 |  | 6 | 64.5 | 9.0 |
| Body mass index | 110 | 26.5 | 5.0 |  | 73 | 26.4 | 5.3 |  | 19 | 26.1 | 4.6 |  | 6 | 22.3 | 3.5 |
| **CPET** |  |  |  |  |  |  |  |  |  |  |  |  |  |  |  |
| VO2peak, ml/kg/min | 110 | 19.1 | 4.0 |  | 73 | 16.3 | 3.7 |  | 19 | 16.1 | 2.2 |  | 6 | 15.9 | 2.3 |
| VO2peak, % predicted | 110 | 90.5 | 14.0 |  | 73 | 72.5 | 11.4 |  | 19 | 76.7 | 7.6 |  | 6 | 56.3 | 3.1 |
| The VE/VCO2 slope | 110 | 30.3 | 4.5 |  | 73 | 35.4 | 2.7 |  | 19 | 46.3 | 4.4 |  | 6 | 43.4 | 2.4 |
| **Spirometry** |  |  |  |  |  |  |  |  |  |  |  |  |  |  |  |
| FEV1, l/min | 110 | 2.1 | 0.7 |  | 73 | 2.1 | 0.6 |  | 19 | 2.1 | 0.6 |  | 6 | 1.8 | 0.5 |
| ppFEV1, % | 110 | 80 | 20 |  | 73 | 72 | 17 |  | 19 | 77 | 17 |  | 6 | 59 | 13 |
| VC, l | 110 | 3.3 | 0.9 |  | 73 | 3.4 | 0.9 |  | 19 | 3.5 | 1.1 |  | 6 | 3.5 | 0.8 |
| ppFVC, % | 110 | 71 | 23 |  | 73 | 72 | 21 |  | 19 | 81 | 27 |  | 6 | 85 | 17 |
| FEV1/VC | 110 | 0.6 | 0.1 |  | 73 | 0.6 | 0.1 |  | 19 | 0.6 | 0.1 |  | 6 | 0.5 | 0.0 |
| DLCOc, mmol/min/kPa | 98 | 5.9 | 1.7 |  | 61 | 5.2 | 1.5 |  | 16 | 4.5 | 1.4 |  | 4 | 3.3 | 0.7 |
| ppDLCOc, % | 98 | 86 | 19 |  | 61 | 69 | 16 |  | 16 | 65 | 17 |  | 4 | 42 | 7 |
| TLC, l | 97 | 6.0 | 1.2 |  | 63 | 6.4 | 1.3 |  | 15 | 6.4 | 1.3 |  | 4 | 7.6 | 1.0 |
| ppTLC, % | 97 | 99 | 14 |  | 63 | 97 | 17 |  | 15 | 98 | 12 |  | 4 | 106 | 9 |
| RV, l | 96 | 2.6 | 0.7 |  | 62 | 2.9 | 0.8 |  | 15 | 2.7 | 0.6 |  | 4 | 3.9 | 0.4 |
| ppRV, % | 96 | 112 | 28 |  | 62 | 122 | 42 |  | 15 | 110 | 23 |  | 4 | 148 | 14 |
|  |  |  |  |  |  |  |  |  |  |  |  |  |  |  |  |
| **Comorbidity** | **N** | **%** |  |  | **N** | **%** |  |  | **N** | **%** |  |  | **N** | **%** |  |
| Coronary artery disease | 13 | 12 |  |  | 11 | 15 |  |  | 4 | 21 |  |  | 1 | 17 |  |
| Previous cardiac surgery | 6 | 6 |  |  | 7 | 10 |  |  | 2 | 11 |  |  | 1 | 17 |  |
| Previous cerebrovascular insult | 7 | 6 |  |  | 6 | 8 |  |  | 1 | 5 |  |  | 2 | 33 |  |
| Current treatment for heart failure | 6 | 6 |  |  | 5 | 7 |  |  | 2 | 11 |  |  | 0 | 0 |  |
| Current treatment for hypertension | 39 | 36 |  |  | 27 | 37 |  |  | 10 | 53 |  |  | 2 | 33 |  |
| Current treatment for arrhythmia | 20 | 18 |  |  | 15 | 21 |  |  | 3 | 16 |  |  | 2 | 33 |  |
| Diabetes mellitus | 20 | 18 |  |  | 15 | 21 |  |  | 3 | 16 |  |  | 2 | 33 |  |
| Chronic kidney disease | 6 | 6 |  |  | 6 | 8 |  |  | 1 | 5 |  |  | 0 | 0 |  |
| Chronic obstructive pulmonary disease | 29 | 26 |  |  | 38 | 52 |  |  | 11 | 60 |  |  | 4 | 67 |  |
|  |  |  |  |  |  |  |  |  |  |  |  |  |  |  |  |
| MITS | 13 | 12 |  |  | 8 | 11 |  |  | 1 | 5 |  |  | 0 | 0 |  |

CPET = cardiopulmonary exercise testing; VCO2 _=_ carbon dioxide elimination; VE= minute ventilation; VO2peak _=_ peak oxygen uptake; FEV1 = forced expiratory volume in 1 second; pp = percent of predicted; VC = vital capacity; FVC = forced vital capacity; DLCOc = diffusing capacity of the lungs for carbon monoxide, corrected for hemoglobin; TLC = total lung capacity; RV = residual volume; MITS = minimally invasive thoracic surgery.

Low risk = < 15% risk, VE/VCO2 slope ≤ 30 or %-VO2 peak > 88%, Intermediate risk = 15-24% risk of complications, VE/VCO2 slope 31-40 and %-VO2 peak ≤ 88 %, High risk = 24-50% risk of complications, VE/VCO2 slope > 40 and %-VO2 peak 62-88%, Very high risk of complications = >50% risk of complications, VE/VCO2 slope > 40 and %-VO2 peak <62%.
