# Supplementary material for: Improved Risk Stratification Prior to Major Pulmonary Resection by Combining Peak Oxygen Uptake and Ventilatory Efficiency in a 9-Field Matrix
Source: CHEST Pulm. 2025 Jul 24;3(4):100192. doi: 10.1016/j.chpulm.2025.100192 (PMC13418083; doi:10.1016/j.chpulm.2025.100192)
Supplement: e-Online Data [file mmc3.pdf]

(A) Lobectomy (n=138)

%-VO2peak		VE/VCO2-slope ≤30 (n=47)	VE/VCO2-slope 31-40 (n=73)	VE/VCO2-slope >40 (n=18)
**>88%**	(n=43)	0/24 (0%)	2/18 (11%)	0/1 (0%)
**62-88%**	(n=81)	2/21 (10%)	9/47 (19%)	5/13 (39%)
**<62%**	(n=14)	0/2 (0%)	2/8 (25%)	3/4 (75%)

P-value 0.006 for difference across cells in matrix

<15 % | 15 - 24 % | 25 - 50 % | >50 %

(B) Pulmectomy (n=20)

%-VO2peak		VE/VCO2-slope ≤30 (n=12)	VE/VCO2-slope 31-40 (n=7)	VE/VCO2-slope >40 (n=1)
**>88%**	(n=4)	0/4 (0%)	NA	NA
**62-88%**	(n=12)	1/7 (14%)	1/5 (20%)	NA
**<62%**	(n=4)	0/1 (0%)	0/2 (0%)	1/1 (100%)

P-value 0.22 for difference across cells in matrix

<15 % | 15 - 24 % | 25 - 50 % | >50 %

(C) Sub-lobular resection (n=50)

%-VO2peak		VE/VCO2-slope ≤30 (n=20)	VE/VCO2-slope 31-40 (n=21)	VE/VCO2-slope >40 (n=9)
**>88%**	(n=21)	1/9 (11%)	2/10 (20%)	0/2 (0%)
**62-88%**	(n=24)	0/11 (0%)	0/7 (0%)	0/6 (0%)
**<62%**	(n=5)	NA	0/4 (0%)	0/1 (0%)

P-value 0.56 for difference across cells in matrix

<15 % | 15 - 24 % | 25 - 50 % | >50 %
